# Joint associations of PM_10_ and smoking with the risk of new-onset stroke in middle-aged and older adult Chinese adults: findings from the CHARLS cohort study

**DOI:** 10.3389/fpubh.2025.1537166

**Published:** 2025-06-02

**Authors:** Shiqin Chen, Tian Lv, Weiyu Li, Liang Yu, Gonghua Pan, Ting Shen

**Affiliations:** ^1^Department of Neurology, Second People's Hospital of Yuhuan, Yuhuan, China; ^2^Department of Neurology, Zhuji Affiliated Hospital of Wenzhou Medical University, Zhuji, China

**Keywords:** particulate matter 10, cigarette smoking, stroke risk, cohort study, environmental exposure

## Abstract

**Background:**

The relationship between long-term exposure to particulate matter ≤ 10 μm in diameter (PM_10_), smoking, and stroke risk remains unclear. This study investigates their association.

**Methods:**

We analyzed data from 10,839 participants in the 2013 wave of the China Health and Retirement Longitudinal Study (CHARLS). Long-term PM_10_ exposure was estimated using the China High Air Pollution (CHAP) dataset, and incident stroke cases were self-reported during follow-up through 2018. Multivariable Cox proportional hazards models, restricted cubic spline (RCS) analyses, and joint exposure models were employed.

**Results:**

Each 1 μg/m^3^ increase in PM_10_ concentration was associated with a 0.3% higher risk of stroke (HR = 1.003; 95% CI: 1.000–1.005; *p* = 0.04). A nonlinear exposure–response relationship was observed (P for non-linearity = 0.04). Among PM_10_ exposure quartiles, only the third quartile (91.90–115.92 μg/m^3^) was significantly associated with increased stroke risk (HR = 1.36; 95% CI: 1.08–1.71; *p* < 0.01). Participants exposed to both high PM_10_ levels (≥91.9 μg/m^3^) and smoking had the highest stroke risk (HR = 1.72; 95% CI: 1.33–2.23; *p* < 0.01). No significant multiplicative or additive interaction between PM_10_ and smoking was found.

**Conclusion:**

Long-term PM_10_ exposure and smoking are independent risk factors for stroke. The elevated risk observed within a specific concentration range of PM_10_ suggests a potential threshold or saturation effect. Individuals exposed to both risk factors are particularly vulnerable, highlighting the need for integrated public health strategies targeting both air quality improvement and smoking cessation.

## 1 Introduction

Stroke is a leading cause of disability and has a high mortality rate among adults. With the aging population, the incidence of stroke continues to rise, placing a substantial economic burden on society (1). Given its severe impact and societal costs, preventing strokes is crucial. Controllable risk factors include hypertension, diabetes, hyperlipidemia, smoking, and obesity, with strategies such as blood pressure reduction, statin therapy, smoking cessation, antiplatelet medications, and dietary modifications proven to reduce stroke risk (1). Additionally, air pollution has emerged as a significant trigger for strokes; it is an omnipresent environmental exposure and a rapidly growing public health threat, particularly in urban areas. Long-term exposure to air pollution has been linked to an increased risk of stroke (2, 3).

Particulate matter ≤ 10 μm in diameter (PM_10_) is a common environmental pollutant primarily generated from coal combustion, road traffic emissions, secondary pollutants, cooking aerosols, and wood smoke (4). This pollutant poses substantial health risks, including impaired lung function (5), increased lung cancer incidence (6), and elevated all-cause mortality rates (7). In addition, PM_10_ exposure is linked to stroke incidence and mortality. Evidence suggests that short-term exposure to elevated PM_10_ levels correlates with increased stroke-related hospital admissions and mortality rates (8, 9). However, the link between long-term PM_10_ exposure and stroke remains inconclusive due to limited research. While a meta-analysis with low to moderate credibility reported no significant association between long-term PM_10_ exposure and stroke risk (10), other studies have shown contrasting results. For instance, a study on older American women found a significant association between prolonged PM_10_ exposure and an elevated risk of cerebrovascular events in postmenopausal women (11). Similarly, findings from the Heinz Nixdorf Recall (HNR) study indicated a positive association between elevated PM_10_ levels and stroke risk in a German cohort (12). These mixed findings underscore the need for further investigation to clarify the impact of long-term PM_10_ exposure on stroke risk.

In contrast, fine particulate matter (PM_2.5_), which is smaller in aerodynamic diameter and can penetrate deeper into the alveolar region and systemic circulation, has been consistently associated with a wide range of adverse health outcomes, including respiratory disease, cardiovascular events, and neurological disorders. Recent evidence summarized in a 2024 review has reaffirmed that long-term PM_2.5_ exposure contributes significantly to the development and progression of stroke through multiple biological pathways such as oxidative stress, systemic inflammation, endothelial dysfunction, and neurovascular damage (13). Moreover, PM_2.5_ contains toxic chemical constituents capable of inducing genotoxicity and epigenetic alterations, further exacerbating cerebrovascular vulnerability (13). These findings suggest that particle size plays a crucial role in determining the health impact of particulate pollution. While PM_2.5_ has been extensively studied, the evidence regarding long-term exposure to PM_10_ remains inconsistent and limited, especially in low- and middle-income countries where PM_10_ levels remain high. As PM_10_ is more coarse and typically coexists with PM_2.5_, nitrogen dioxide (NO₂), and ozone (O₃), understanding its independent and joint effects with other pollutants is essential to clarify its contribution to stroke risk.

Smoking is a well-established risk factor for stroke, with harmful substances inhaled directly through the respiratory tract. The amount of smoking, measured in pack-years, is significantly associated with stroke risk, accounting for nearly 15% of stroke-related deaths each year (1). Tobacco smoke and air pollutants such as PM_10_ share overlapping exposure routes and pathogenic mechanisms, including oxidative damage and vascular inflammation (14). Despite this, few studies have assessed the joint effects of long-term PM_10_ exposure and smoking on stroke risk. This study aims to use the China Health and Retirement Longitudinal Study (CHARLS) data to independently examine the association between long-term PM_10_ exposure and stroke risk, followed by a joint analysis of PM_10_, smoking, and stroke risk. We hypothesize that high levels of long-term PM_10_ exposure, combined with smoking, will elevate stroke risk.

## 2 Methods

### 2.1 Study population

This study was a secondary analysis using data from CHARLS, a longitudinal study of Chinese adults aged 45 and older. Data were collected at both the household and individual levels. The baseline survey in 2011 sampled 150 counties or districts across 450 villages or neighborhoods, with follow-up waves conducted every 2–3 years (Wave 1 in 2011, Wave 2 in 2013, Wave 3 in 2015, and Wave 4 in 2018) (15).

**Figure 1:**
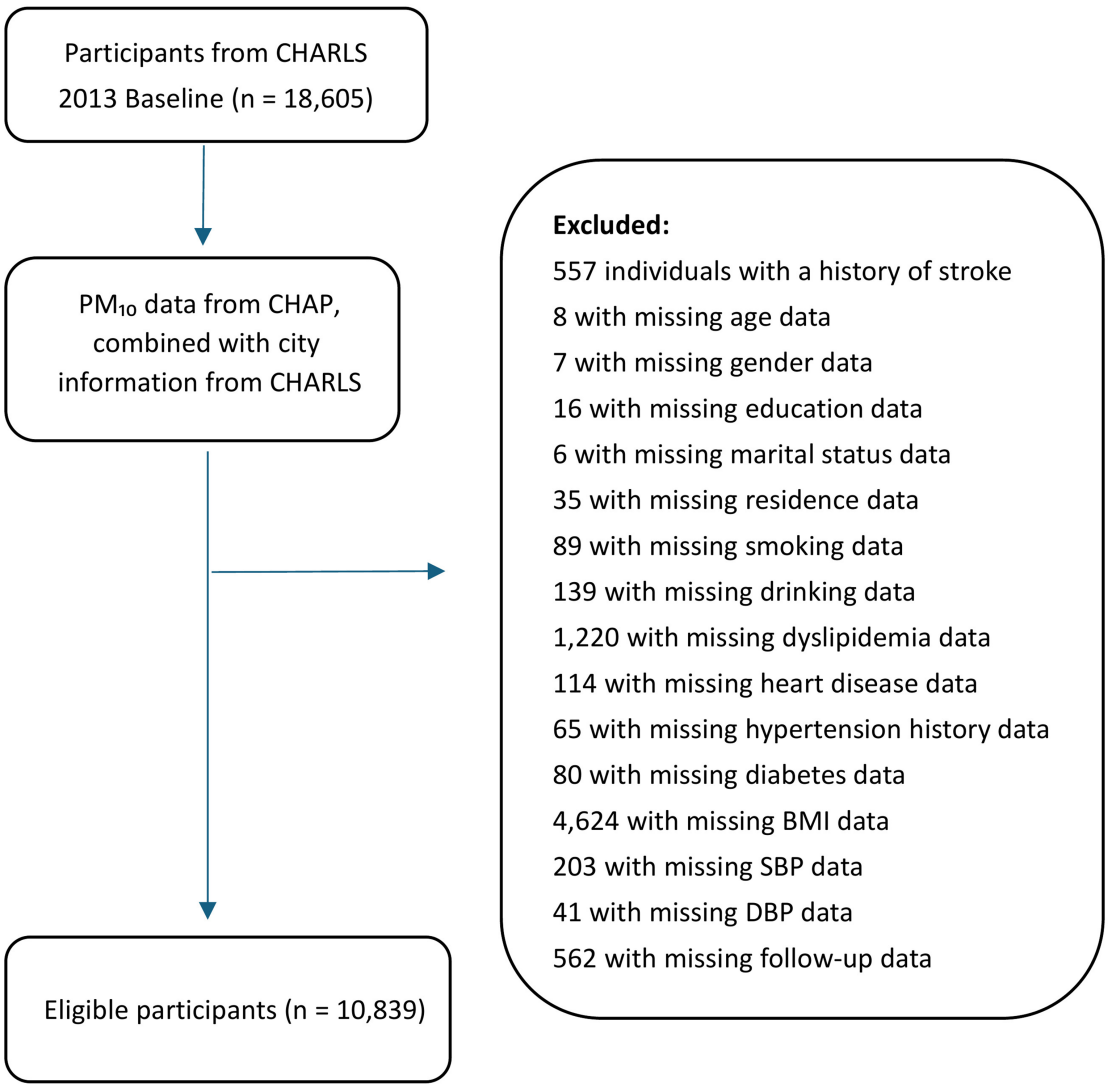
Flowchart of study inclusion and exclusion criteria. CHARLS, China Health and Retirement Longitudinal Study; CHAP, China High Air Pollution; PM_10_, particulate matter ≤ 10 μm in diameter; BMI, body mass index; SBP, systolic blood pressure; DBP, diastolic blood pressure.

For this analysis, we used the CHARLS 2013 cohort as the baseline population, including 18,605 individuals, with a 5-year follow-up period. Participants were eligible if they had complete demographic, health, and physical examination data and no history of stroke at baseline. A total of 10,839 participants met these inclusion criteria. The study design flowchart is shown in Figure 1.

### 2.2 Long-term PM_10_ exposure data

High-quality, high-resolution PM_10_ data for each participant were sourced from the China High Air Pollution (CHAP) dataset. Wei et al. generated this high-accuracy, 1-kilometer resolution dataset using a space–time extremely randomized trees (STET) model, which incorporated data from the Multi-Angle Implementation of Atmospheric Correction (MAIAC) aerosol products, along with meteorological conditions, surface elevation, land use cover, and pollutant emissions (16). This dataset has been widely used in research exploring the effects of particulate pollution on health and cognitive outcomes among older populations (17, 18). In our primary analysis, we used the average PM_10_ concentration in 2013 as the baseline for assessing long-term exposure. Although Wave 1 of CHARLS enrolled participants in 2011 and 2012, we selected Wave 2 (conducted exclusively in 2013) as the baseline to ensure temporal consistency with the exposure data. The CHAP dataset provides high-resolution annual PM_10_ data from 2000 to 2023, and using the 2013 data aligned the exposure assessment with the timing of participant follow-up while avoiding variability associated with the multi-year enrollment in Wave 1.

### 2.3 Smoking behavior

Smoking is recognized as an addictive behavior by the World Health Organization’s 2003 Framework Convention on Tobacco Control (19). In our study, we used health-related variables from CHARLS along with participants’ self-reported smoking status. Smoking status was categorized into two groups: non-smoker and smoker (20).

### 2.4 Primary endpoint

The primary endpoint in this study was defined as the initial occurrence of a stroke. Stroke events were identified based on participants’ responses to specific survey questions, such as: “Has a doctor ever diagnosed you with a stroke?”; “Since your last follow-up, has a doctor diagnosed you with a stroke?”; and “Did an interview with a knowledgeable informant confirm a stroke diagnosis?” The timing of stroke events was gathered through questions like: “When did you first become aware of or receive a stroke diagnosis?”; “What was the date of your last stroke diagnosis?”; and “In the exit interview following death, what year was the latest stroke diagnosis noted?” Participants were followed from 2013 through three interview waves until the first occurrence of stroke or until the end of 2018, whichever came first.

### 2.5 Covariates

In the 2013 Wave 2 data, trained interviewers collected sociodemographic, health, and physical examination data through a structured questionnaire. Demographic variables included age, gender (male, female), marital status (married, unmarried), residence type (rural, urban), and educational level (categorized as below secondary or secondary and above), based on recent studies (21, 22). Health-related variables encompassed self-reported alcohol consumption status (categorized as never or ever), self-reported physician-diagnosed dyslipidemia (yes, no), self-reported physician-diagnosed heart disease (yes, no), and self-reported history of hypertension and diabetes (yes, no). Physical examination variables included blood pressure (systolic and diastolic), measured three times and averaged using the Omron HEM-7200 blood pressure monitor, and body mass index (BMI), calculated as weight/height^2^ (kg/m^2^).

### 2.6 Statistical analysis

Continuous variables are presented as means and standard deviations (SD). Between-group comparisons were performed using the Student’s *t*-test or Mann–Whitney *U* test, depending on the data distribution. Categorical variables are reported as frequencies and percentages, with between-group comparisons performed using the chi-square test.

To assess the association between PM_10_ levels and stroke risk, we employed Cox proportional hazards regression models. Sensitivity analyses included three models: Model 1 with no covariate adjustment, Model 2 adjusted for demographic factors (age, gender, education, marital status, and residence), and Model 3 further adjusted for health factors [smoking, drinking, dyslipidemia, heart problems, hypertension, diabetes, BMI, systolic blood pressure (SBP), and diastolic blood pressure (DBP)] beyond Model 2. We used restricted cubic splines (RCS) to explore potential nonlinear associations between PM_10_ exposure and stroke risk. Additional stratified analyses were performed by categorizing PM_10_ levels into quartiles, adjusting for the same covariates across the three models for sensitivity testing.

For the joint analysis of PM_10_, smoking, and stroke risk, participants were divided into four groups based on the median PM_10_ concentration (91.9 μg/m^3^) and smoking status: (1) PM_10_ < 91.9 μg/m^3^ and non-smoker, (2) PM_10_ < 91.9 μg/m^3^ and smoker, (3) PM_10_ ≥ 91.9 μg/m^3^ and non-smoker, and (4) PM_10_ ≥ 91.9 μg/m^3^ and smoker. Three adjusted models were applied, with Model 3 excluding smoking in the joint analysis. To explore potential interactions between PM_10_ levels and smoking, we performed an interaction analysis in a Cox model using the PM_10_*smoking term. We also analyzed the additive interaction between PM_10_ and smoking using the “interactionR” R package. Subgroup analyses by age (< 60, ≥ 60), gender, and education level were conducted to examine stroke risk across different population groups, with the same adjustments applied for sensitivity analysis.

All analyses were conducted using R software version 4.4.1, with a two-sided *p*-value < 0.05 considered statistically significant.

## 3 Results

### 3.1 Participant characteristics

A total of 10,839 participants were included in the final analysis after excluding 557 individuals with a baseline history of stroke, 72 with missing demographic data, 1,707 with missing health status data, 4,868 with missing physical examination data, and 562 with missing follow-up information. The mean age of the participants was 59.7 years, and women comprised 53.5% of the sample. Among the participants, 68.5% had an education level below secondary school, and 41.4% were smokers. The average long-term PM_10_ exposure concentration was 98.3 μg/m^3^. Detailed participant characteristics are shown in Table 1.

**Table 1:** Baseline characteristics of participants according to stroke status (*N* = 10,839).

**Characteristic**		**Overall**	**Contrl**	**Stroke**	***p*-value**
*N*		10,839	10,126	713	
Age (Mean (Sd))		59.7 (9.6)	59.5 (9.7)	63.3 (8.1)	<0.01
Gender (%)	Female	5,798 (53.5)	5,428 (53.6)	370 (51.9)	0.40
	Male	5,041 (46.5)	4,698 (46.4)	343 (48.1)	
Education (%)	Below secondary	7,422 (68.5)	6,894 (68.1)	528 (74.1)	<0.01
	Secondary or above	3,417 (31.5)	3,232 (31.9)	185 (25.9)	
Marital (%)	Married	9,535 (88.0)	8,926 (88.1)	609 (85.4)	0.04
	Unmarried	1,304 (12.0)	1,200 (11.9)	104 (14.6)	
Residence (%)	Rural	8,715 (80.4)	8,144 (80.4)	571 (80.1)	0.86
	Urban	2,124 (19.6)	1982 (19.6)	142 (19.9)	
Smoking (%)	No	6,351 (58.6)	5,963 (58.9)	388 (54.4)	0.02
	Yes	4,488 (41.4)	4,163 (41.1)	325 (45.6)	
Drinking (%)	None	6,028 (55.6)	5,631 (55.6)	397 (55.7)	1.00
	Yes	4,811 (44.4)	4,495 (44.4)	316 (44.3)	
Dyslipidemia (%)	No	9,527 (87.9)	8,979 (88.7)	548 (76.9)	<0.01
	Yes	1,312 (12.1)	1,147 (11.3)	165 (23.1)	
Heart_problem (%)	No	9,460 (87.3)	8,924 (88.1)	536 (75.2)	<0.01
	Yes	1,379 (12.7)	1,202 (11.9)	177 (24.8)	
Hypertension (%)	No	7,991 (73.7)	7,620 (75.3)	371 (52.0)	<0.01
	Yes	2,848 (26.3)	2,506 (24.7)	342 (48.0)	
Diabetes (%)	No	10,120 (93.4)	9,490 (93.7)	630 (88.4)	<0.01
	Yes	719 (6.6)	636 (6.3)	83 (11.6)	
BMI (Mean (Sd))		23.9 (3.8)	23.8 (3.7)	24.6 (4.1)	<0.01
SBP (Mean (Sd))		130.1 (21.1)	129.6 (20.9)	137.9 (22.7)	<0.01
DBP (Mean (Sd))		76.4 (12.7)	76.2 (12.6)	79.6 (13.2)	<0.01
PM₁₀ (Mean (Sd))		98.3 (30.5)	98.0 (30.5)	102.4 (29.8)	<0.01
PM₁₀_quat (%)	Q1 (54.54–75.76 μg/m^3^)	2,269 (20.9)	2,149 (21.2)	120 (16.8)	<0.01
	Q2 (75.76–91.90 μg/m^3^)	2,924 (27.0)	2,771 (27.4)	153 (21.5)	
	Q3 (91.90–115.92 μg/m^3^)	2,634 (24.3)	2,415 (23.8)	219 (30.7)	
	Q4 (115.92–179.35 μg/m^3^)	3,012 (27.8)	2,791 (27.6)	221 (31.0)	
Joint (%)	PM₁₀ < 91.9 ug/m^3^ and non-Smoker	3,097 (28.6)	2,947 (29.1)	150 (21.0)	<0.01
	PM₁₀ < 91.9 ug/m^3^ and smoker	2096 (19.3)	1973 (19.5)	123 (17.3)	
	PM₁₀ > = 91.9 ug/m^3^ and non-Smoker	3,254 (30.0)	3,016 (29.8)	238 (33.4)	
	PM₁₀ > = 91.9 ug/m^3^ and smoker	2,392 (22.1)	2,190 (21.6)	202 (28.3)	

BMI, body mass index; SBP, systolic blood pressure; DBP, diastolic blood pressure; PM_10_, particulate matter ≤ 10 μm in diameter; PM_10__quat, quartiles of PM_10_ exposure.

### 3.2 Association between PM_10_ and stroke risk

Table 2 presents the results of the three Cox regression models analyzing the association between PM_10_ levels and stroke risk. In Model 3, after adjusting for age, gender, education, marital status, residence, smoking, drinking, dyslipidemia, heart problems, hypertension, diabetes, BMI, SBP, and DBP, a positive association was observed between PM_10_ concentration and stroke risk (HR = 1.003, 95% CI: 1.000–1.005; *p* = 0.04).

**Table 2:** Sensitivity analyses of the association between PM_10_ exposure and stroke risk.

Variable	Model1 HR(95% CI)	*p*-value	Model2 HR(95% CI)	*p*-value	Model3 HR(95% CI)	*p*-value
PM₁₀	1.004 (1.002, 1.006)	<0.01	1.005 (1.003, 1.007)	<0.01	1.003 (1.000, 1.005)	0.04
PM₁₀_quatQ1 (54.54–75.76 μg/m^3^)	Ref		Ref		Ref	
PM₁₀_quatQ2 (75.76–91.90 μg/m^3^)	0.983 (0.774, 1.248)	0.89	0.939 (0.739, 1.192)	0.60	0.915 (0.719, 1.163)	0.47
PM₁₀_quatQ3 (91.90–115.92 μg/m^3^)	1.578 (1.263, 1.972)	< 0.01	1.633 (1.306, 2.041)	< 0.01	1.358 (1.081, 1.705)	< 0.01
PM₁₀_quatQ4 (115.92–179.35 μg/m^3^)	1.363 (1.091, 1.702)	< 0.01	1.440 (1.152, 1.800)	< 0.01	1.182 (0.941, 1.486)	0.15

Model 1: Unadjusted. Model 2: Adjusted for age, gender, education, marital status, and residence. Model 3: Additionally adjusted for smoking, drinking, dyslipidemia, heart conditions, hypertension, diabetes, body mass index, systolic blood pressure, and diastolic blood pressure. PM_10_, Particulate Matter ≤ 10 μm in diameter; PM_10__quat, Quartiles of PM_10_ exposure; HR, Hazard Ratio; CI, Confidence Interval.

**Figure 2:**
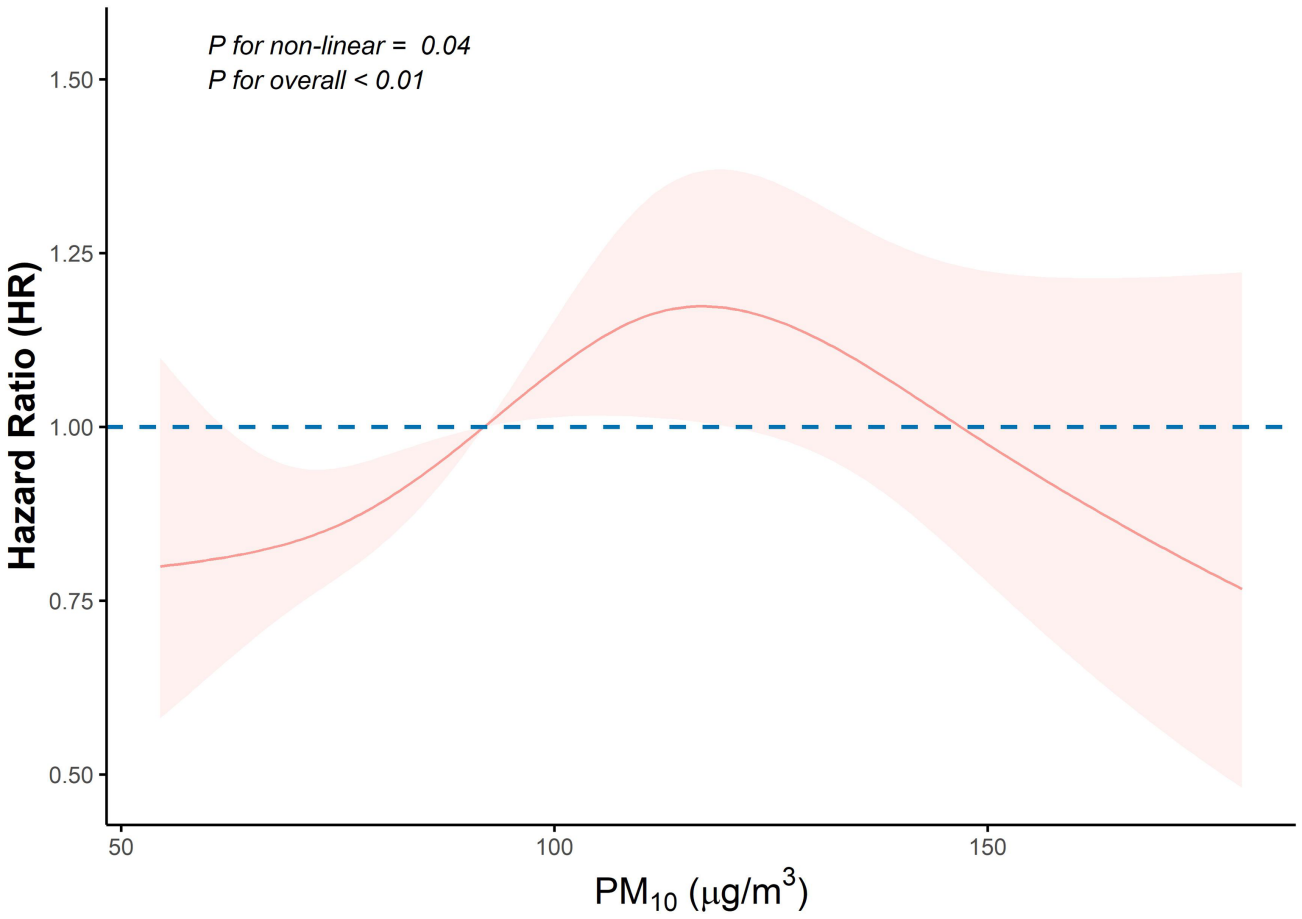
Nonlinear association between PM_10_ exposure and stroke risk. The restricted cubic spline curve shows the adjusted hazard ratio (HR) of stroke across PM_10_ concentrations. The model adjusted for age, gender, education, marital status, residence, smoking, drinking, dyslipidemia, heart conditions, hypertension, diabetes, body mass index, systolic blood pressure, and diastolic blood pressure. The dashed horizontal line indicates the reference (HR = 1.0). *p*-values test the nonlinearity (*p* = 0.04) and overall association (*p* < 0.01). Shaded regions represent 95% confidence intervals. PM_10_, particulate matter ≤ 10 μm in diameter.

Further, an adjusted restricted cubic spline (RCS) regression analysis showed a nonlinear association between PM_10_ concentration and stroke risk (P for non-linearity = 0.04; Figure 2). In a stratified analysis by PM_10_ quartiles (also adjusted using Model 3), Quartile 3 was significantly associated with an increased stroke risk compared to the lowest PM_10_ quartile (Quartile 1) (HR = 1.36, 95% CI: 1.08–1.71; *p* < 0.01). No statistically significant differences were found for the other quartiles.

### 3.3 Joint analysis of PM_10_ and smoking with stroke risk

**Figure 3:**
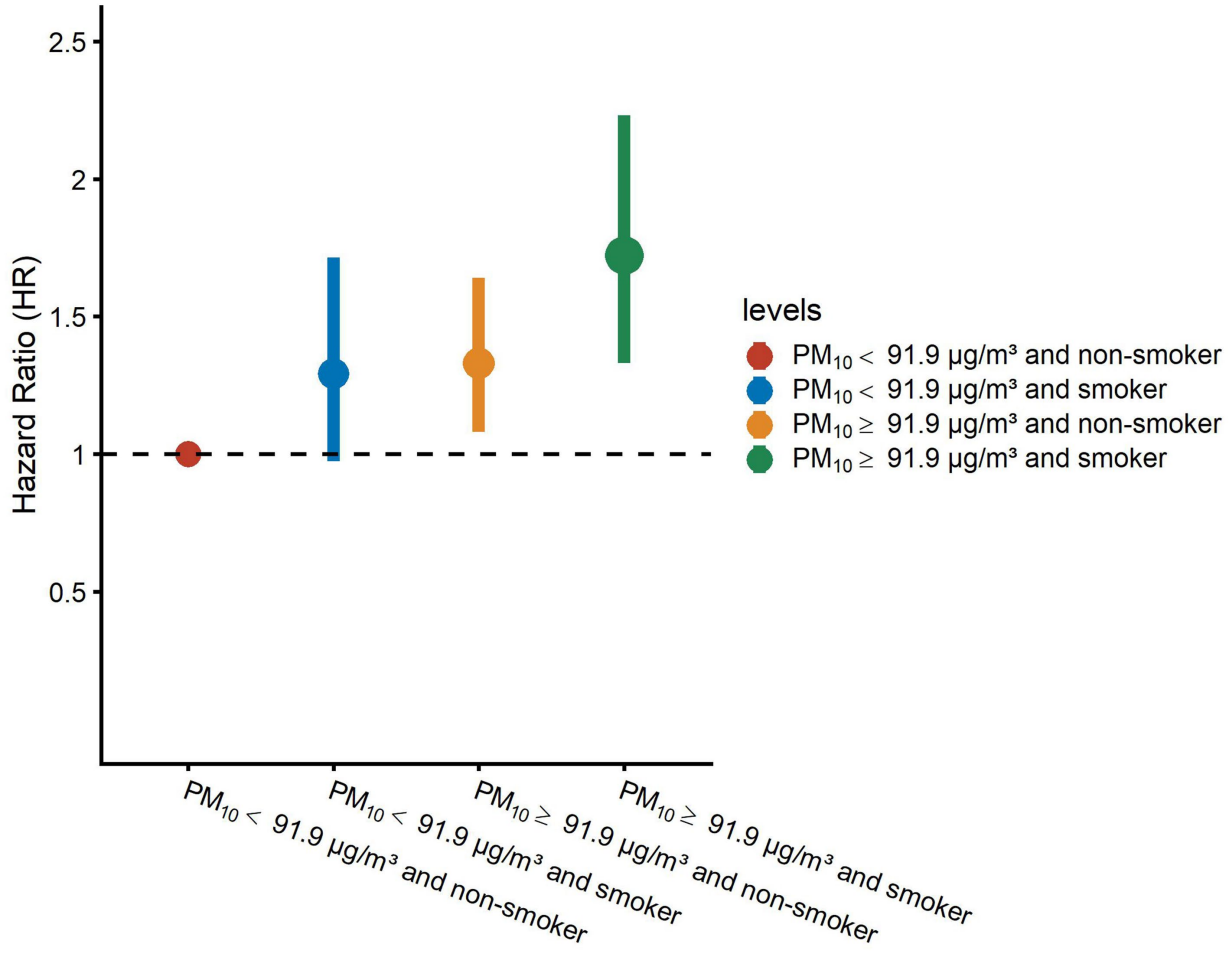
Hazard ratios (HRs) of stroke risk by PM_10_ exposure and smoking status. Adjusted hazard ratios (dots) and 95% confidence intervals (bars) show stroke risk across combined categories of PM_10_ exposure (stratified at 91.9 μg/m^3^) and smoking status. The model was adjusted for age, gender, education, marital status, residence, drinking, dyslipidemia, heart conditions, hypertension, diabetes, body mass index, systolic blood pressure, and diastolic blood pressure. PM_10_, particulate matter ≤ 10 μm in diameter.

Table 3 presents the results from the joint analysis of PM_10_ levels and smoking status on stroke risk using three Cox models. In Model 3, which adjusted for age, gender, education, marital status, residence, drinking, dyslipidemia, heart problems, hypertension, diabetes, BMI, SBP, and DBP (excluding smoking), we observed that participants with PM_10_ ≥ 91.9 μg/m^3^ who were also smokers had the highest stroke risk (HR = 1.72, 95% CI: 1.33–2.23; *p* < 0.01) compared to the reference group (PM_10_ < 91.9 μg/m^3^ and non-smokers). Additionally, non-smokers with PM_10_ ≥ 91.9 μg/m^3^ also exhibited an elevated stroke risk (HR = 1.33, 95% CI: 1.08–1.64; *p* < 0.01). However, the group with PM_10_ < 91.9 μg/m^3^ who were smokers did not show a statistically significant increase in stroke risk. To clarify these comparisons, the results have been visualized in Figure 3 for easier interpretation.

**Table 3:** Sensitivity analyses of the joint association of variables on stroke risk.

Variable	Model1 HR (95% CI)	*p-*value	Model2 HR (95% CI)	*p-*value	Model3 HR (95% CI)	*p-*value
PM₁₀ < 91.9 μg/m^3^ and non-smoker	Ref		Ref		Ref	
PM₁₀ < 91.9 μg/m^3^ and smoker	1.25 (0.99, 1.59)	0.06	1.25 (0.94, 1.65)	0.13	1.29 (0.97, 1.72)	0.08
PM₁₀ > = 91.9 μg/m^3^ and non-smoker	1.50 (1.22, 1.84)	< 0.01	1.58 (1.29, 1.94)	< 0.01	1.33 (1.08, 1.64)	< 0.01
PM₁₀ > = 91.9 μg/m^3^ and smoker	1.80 (1.46, 2.23)	< 0.01	1.96 (1.52, 2.53)	< 0.01	1.72 (1.33, 2.23)	< 0.01
Interaction						
PM₁₀ * Smoking	1.00 (0.99, 1.00)	0.38	1.00 (0.99, 1.00)	0.49	1.00 (0.99, 1.00)	0.69

Model 1: Unadjusted. Model 2: Adjusted for age, gender, education, marital status, and residence. Model 3: Additionally adjusted for drinking, dyslipidemia, heart conditions, hypertension, diabetes, body mass index, systolic blood pressure, and diastolic blood pressure. PM_10_, Particulate Matter ≤ 10 μm in diameter; HR, Hazard Ratio; CI, Confidence Interval.

We further evaluated the interaction between PM_10_ and smoking. In the adjusted Model 3, analysis of the PM_10_*smoking interaction term revealed no significant interaction (*p* > 0.05) (Table 3). Additionally, Table 4 shows no additive interaction between PM_10_ and smoking.

**Table 4:** Interaction between PM_10_ exposure and smoking on stroke risk.

PM_10_ Level / Smoking Status	Non-smoker	Smoker	Effect of smoking within the strata of PM_10_ > = 91.9 μg/m^3^
	OR [95% CI]	OR [95% CI]	OR [95% CI]
PM_10_ < 91.9 μg/m^3^	1 [Reference]	1.29 [0.97, 1.71]	1.29 [0.97, 1.71]
PM_10_ > = 91.9 μg/m^3^	1.33 [1.08, 1.64]	1.72 [1.33, 2.23]	1.29 [1.02, 1.65]
Effect of PM_10_ > = 91.9 μg/m^3^ within the strata of smoking	1.33 [1.08, 1.64]	1.33 [1.06, 1.68]	
Delta method			
Interaction Measure	Estimate [95% CI]		
Multiplicative scale	1 [0.74, 1.36]		
RERI	0.1 [−0.3, 0.5]		
AP	0.06 [−0.17, 0.29]		
SI	1.16 [0.61, 2.19]		

Interaction was assessed on the additive scale using RERI, AP, and SI. PM_10_, Particulate Matter ≤ 10 μm in diameter; RERI, Relative Excess Risk due to Interaction; AP, Attributable Proportion; SI, Synergy Index; OR, Odds Ratio; CI, Confidence Interval. Although the interaction analysis was based on a Cox model, the interactionR package estimates additive interaction measures using odds ratios. The 95% confidence intervals for RERI and AP include 0, and for SI, the 95% confidence interval includes 1, indicating no significant additive interaction between PM_10_ exposure and smoking on stroke risk.

### 3.4 Subgroup analysis

**Figure 4:**
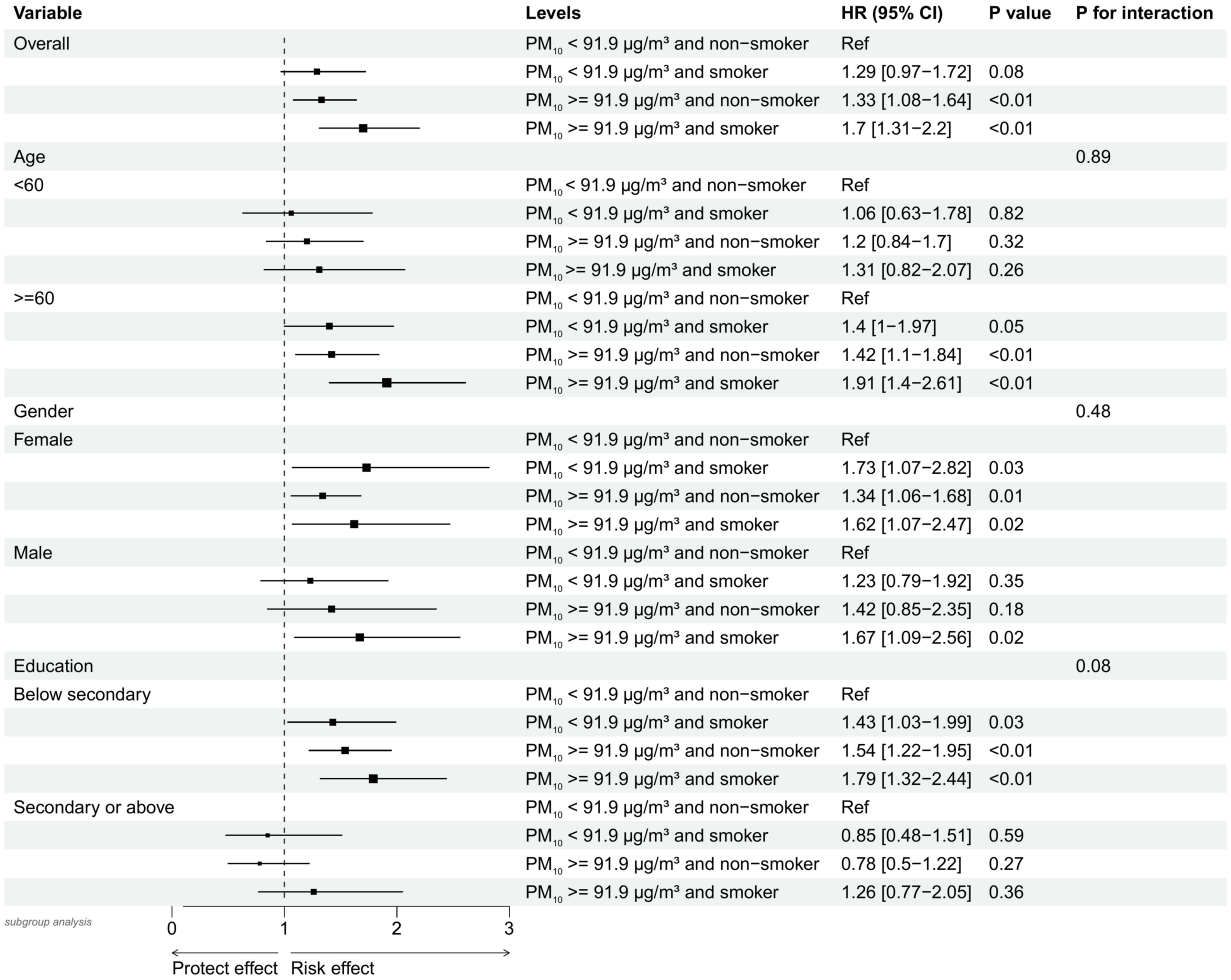
Subgroup analysis of stroke risk by combined PM_10_ exposure and smoking status, stratified by age, gender, and education level Forest plot showing hazard ratios (HRs) and 95% confidence intervals (CIs) for stroke risk in subgroups defined by combined PM_10_ exposure (cutoff: 91.9 μg/m^3^) and smoking status. Analyses are stratified by age (<60 vs. ≥60 years), gender, and education level. Reference group (Ref): non-smokers with PM_10_ exposure below 91.9 μg/m^3^. PM_10_, Particulate Matter ≤ 10 μm in diameter; HR, Hazard Ratio.

Figure 4 presents the results of subgroup analyses stratified by age, gender, and educational attainment. After adjusting for age, gender, education, marital status, residence, drinking, dyslipidemia, heart problems, hypertension, diabetes, BMI, SBP, and DBP, we found that stroke risk was significantly elevated among participants aged ≥ 60 years with PM_10_ levels ≥ 91.9 μg/m^3^ who were smokers, compared to the reference group (PM_10_ < 91.9 μg/m^3^ and non-smokers), with a hazard ratio (HR) of 1.91 (95% CI: 1.40–2.61; *p* < 0.01).

Similarly, individuals with education levels below secondary school and PM_10_ exposure ≥ 91.9 μg/m^3^ who were smokers also showed a significantly increased stroke risk (HR = 1.79, 95% CI: 1.32–2.44; *p* < 0.01). However, no statistically significant difference in stroke risk was observed between male and female participants in this analysis.

## 4 Discussion

This study investigated the association between PM_10_ exposure, smoking, and stroke risk. We found a non-linear, positive relationship between PM_10_ levels and stroke risk. Additionally, joint analysis of PM_10_ exposure and smoking showed that individuals exposed to high PM_10_ levels (≥ 91.9 μg/m^3^) and who smoked had the highest stroke risk compared to the reference group (PM_10_ < 91.9 μg/m^3^ and non-smokers). These findings indicate that long-term exposure to elevated levels of inhalable particulate matter (PM_10_), combined with smoking, markedly increases stroke risk.

Our study found a positive association between long-term exposure to PM_10_ and stroke risk. Although this association was statistically significant (HR = 1.003), the effect size was minimal. Currently, there is no universally accepted minimal clinically important difference (MCID) for stroke risk related to air pollution. Nevertheless, the effect size observed in our study aligns with findings from previous epidemiological research. As noted in the American Heart Association’s scientific statement by Brook et al. (23), even small increases in cardiovascular risk due to air pollution can have meaningful public health consequences, given the large number of individuals exposed (23). Supporting this, a recent meta-analysis by Lin et al. (24) reported a 0.6% increase in stroke incidence for every 10 μg/m^3^ rise in PM_10_ (OR = 1.006, 95% CI: 1.004–1.009), a magnitude comparable to that found in our study (24). We also observed a significant association in Quartile 3 (Q3) of PM_10_ exposure, but not in Quartile 4 (Q4). Several factors may account for this non-monotonic pattern. One possibility is that Q4 had fewer stroke cases or participants, or that participants differed in demographic or behavioral characteristics compared to those in Q3. Prior studies have shown that such imbalances can influence the stability and detectability of associations (25). Another explanation is the presence of a threshold, beyond which additional PM_10_ exposure does not further increase stroke risk. Alternatively, physiological adaptations at higher exposure levels may reduce susceptibility. Similar nonlinear or threshold effects have been reported in other studies (26, 27).

Long-term exposure to PM_10_ has also been linked to increased stroke mortality. A multi-center study of 22 European cohort studies found that higher levels of both PM_10_ and PM_2.5_ were associated with increased mortality from cerebrovascular diseases (28). Although the exact pathophysiological mechanisms underlying the effects of long-term PM_10_ exposure on stroke risk remain unclear, substantial evidence suggests that prolonged exposure to high levels of PM_10_ and PM_2.5_ increases systemic inflammation and thrombosis markers (29–32). Interestingly, research by Adami et al. found that long-term exposure to elevated PM_10_ levels is associated with several autoimmune-mediated diseases (33). These studies suggest that PM_10_ exposure may involve complex inflammatory, coagulation, and immune processes, which contribute to stroke risk and mortality. Further research is needed to clarify these underlying mechanisms.

Smoking is a significant risk factor for stroke. Tobacco smoke is a major source of indoor PM pollution; for example, Invernizzi et al. found that PM_10_ and PM_2.5_ emissions from cigarettes in certain settings can exceed those from diesel engines by more than 10 times (34). Additionally, PM and cigarette smoke may share similar pathophysiological mechanisms, potentially resulting in complementary or synergistic effects. Smokers exposed to PM_2.5_ have a higher relative risk of heart disease and hypertension than non-smokers (35). While no studies to date specifically examine the combined impact of long-term PM_10_ exposure and smoking on stroke risk, research on other diseases, such as pneumonia, has shown a strong synergistic effect, with significantly increased pneumonia risk among smokers exposed to air pollution (36). In our study, the highest stroke risk was observed among individuals exposed to PM_10_ levels ≥ 91.9 μg/m^3^ who also smoked, compared to those exposed to PM_10_ < 91.9 μg/m^3^ and who did not smoke. However, interaction analysis revealed no statistically significant interaction between PM_10_ and smoking, suggesting that each factor independently contributes to an increased risk of stroke. Additionally, subgroup analysis showed a significantly elevated stroke risk among participants aged ≥ 60 and those with education below the secondary level when exposed to PM_10_ levels ≥ 91.9 μg/m^3^ and who smoked. The heightened susceptibility among older adults may be attributed to age-related physiological changes, such as reduced pulmonary clearance, increased systemic inflammation, endothelial dysfunction, and higher prevalence of comorbidities, which can amplify the deleterious effects of air pollutants and tobacco smoke on cerebrovascular health (23, 37). Furthermore, individuals with lower educational attainment may face multiple structural and behavioral disadvantages, including lower health literacy, poorer access to healthcare, and a higher likelihood of residing in more polluted areas, which can collectively exacerbate the impact of environmental and behavioral risk factors (38, 39).

These findings are essential for shaping environmental health policies, clinical practices, and stroke risk stratification efforts. Identifying high-risk individuals by evaluating combined PM_10_ levels and smoking status can support personalized prevention strategies. For instance, adults aged 60 and above who smoke and are exposed to high PM_10_ levels are at an increased risk of stroke, emphasizing the need for proactive management of these risk factors. This approach is particularly important for implementing public health strategies to reduce stroke risk in areas with poor air quality. Additionally, environmental health professionals should closely monitor regional PM_10_ levels, aiming to maintain them below 91.9 μg/m^3^ or lower.

This study has several strengths, including its prospective design, the joint analysis of PM_10_ and smoking on stroke risk, and a large sample size (10,839 participants), which provides sufficient statistical power. However, several limitations should be acknowledged. First, stroke diagnoses were based on self-reported questionnaires, which may be subject to misclassification bias, including both false positives and false negatives. Second, smoking status was dichotomized as “smoker” versus “non-smoker,” without information on smoking intensity or duration, which may lead to residual confounding and underestimation of the heterogeneity in smoking-related stroke risk. Third, only PM_10_ data from 2013 were available and used as a proxy for long-term exposure; this approach may not capture temporal variability in exposure and could result in exposure misclassification. Fourth, as with most observational studies, there remains the possibility of residual confounding due to unmeasured or incompletely measured variables. Fifth, the exclusion of individuals with missing medical examination data may have introduced selection bias and potentially underestimated the association between PM_10_, smoking, and stroke risk. Finally, the study population consisted exclusively of Chinese (Asian) participants, which may limit the generalizability of our findings to other ethnic groups.

## 5 Conclusion

In this nationally representative cohort of middle-aged and older adult Chinese adults, we found that long-term exposure to PM_10_ and smoking were independently associated with an increased risk of new-onset stroke. Notably, the risk was significantly elevated within a specific PM_10_ concentration range (91.90–115.92 μg/m^3^), indicating a possible threshold or saturation effect in the exposure–response relationship. Furthermore, individuals exposed to both high PM_10_ levels and smoking demonstrated the highest susceptibility to stroke, although no significant interaction between the two factors was detected. Subgroup analyses further confirmed the robustness of these associations, particularly among older adults and those with lower educational attainment. These findings underscore the importance of coordinated public health interventions aimed at reducing air pollution and promoting smoking cessation to mitigate stroke risk, especially in vulnerable populations.

## Data Availability

Publicly available datasets were analyzed in this study. This data can be found here: The dataset used in this study was publicly available and can be accessed at http://charls.pku.edu.cn/en and https://weijing-rs.github.io/product.html. The data generated from the analysis can be obtained from the corresponding author upon request.
